# Degree of functionalisation dependence of individual Raman intensities in covalent graphene derivatives

**DOI:** 10.1038/srep45165

**Published:** 2017-03-27

**Authors:** Philipp Vecera, Siegfried Eigler, Maria Koleśnik-Gray, Vojislav Krstić, Asmus Vierck, Janina Maultzsch, Ricarda A. Schäfer, Frank Hauke, Andreas Hirsch

**Affiliations:** 1 Department of Chemistry and Pharmacy and Joint Institute of Advanced Materials and Processes (ZMP), Friedrich-Alexander-Universität Erlangen-Nürnberg (FAU), Henkestrasse 42, 91054 Erlangen, Germany; 2 Chair for Applied Physics, Department of Physics, Friedrich-Alexander-Universität Erlangen-Nürnberg (FAU), Staudtstraße 7, 91058 Erlangen, Germany; 3 Institut für Festkörperphysik, Technische Universität Berlin, Eugene-Wigner-Building EW 5-4, Hardenbergstrasse 36, 10623 Berlin, Germany

## Abstract

Covalent functionalisation of graphene is a continuously progressing field of research. The optical properties of such derivatives attract particular attention. In virtually all optical responses, however, an enhancement in peak intensity with increase of sp^3^ carbon content, and a vanishing of the peak position shift in monolayer compared to few-layer systems, is observed. The understanding of these seemingly connected phenomena is lacking. Here we demonstrate, using Raman spectroscopy and *in situ* electrostatic doping techniques, that the intensity is directly modulated by an additional contribution from photoluminescent *π*-conjugated domains surrounded by sp^3^ carbon regions in graphene monolayers. The findings are further underpinned by a model which correlates the individual Raman mode intensities to the degree of functionalisation. We also show that the position shift in the spectra of solvent-based and powdered functionalised graphene derivatives originates predominantly from the presence of edge-to-edge and edge-to-basal plane interactions and is by large functionalisation independent.

The optical properties of chemically functionalised graphene derivatives^1^,^2^,^3^,^4^,^5^,^6^,^7^,^8^,^9^,^10^ provide a platform with a broad range of possibilities for producing novel technologically relevant 2D carbon-based materials^11^,^12^,^13^. The absorption^1^,^2^, transmission^3^, fluorescence^4^,^5^, photoluminescence^1^,^2^,^6^,^7^ and Raman^8^,^9^,^10^ spectra of covalently functionalised graphene have been found to depend on the degree of functionalisation *θ,* defined as the ratio of C(sp^3^) to C(sp^2^) content in the material^14^. In particular, a significant intensity enhancement in the response signal with *θ* has been observed in various functionalised graphene derivatives^1^,^2^,^7^,^8^,^15^. Furthermore, a characteristic shift of the peak positions with *θ* was reported in solvent-based^1^,^16^,^17^ and bulk (powdered) functionalised graphene^18^, while the positions of the individual peaks remained constant for restacked monolayer derivatives^1^. The origin of both of the phenomena, and whether or not the two are connected, is under general debate. Moreover, the aforementioned spectroscopic methods are powerful tools not only for analysing the optical properties of carbon-based materials^19^; they also provide the operational range for graphene in potential optoelectronic devices^20^.

The introduction of chemical moieties in graphene leads to an sp^2^-to-sp^3^ rehybridization of lattice carbon atoms and the formation of *π*-conjugated domains of varying shapes and sizes^6^,^21^. Within the localized sp^2^ domains local bandgaps are predicted^21^ with energies depending on the domain area^21^. Such domains are generally considered to be carbon-based photoluminescent centres^22^,^23^ and can be expected to have a strong impact on the interaction between light and graphene derivatives.

We selected Raman spectroscopy, including a combined *in situ* Raman-spectroscopy and dry electrostatic doping method, as a principally non-invasive method for probing the (localized) sp^2^ areas of differently functionalised graphene derivatives^24^,^25^,^26^,^27^. Here we show that for monolayer functionalised graphene, the increase in optical response with *θ* is directly associated with the presence of photoluminescent C(sp^2^) domains in the material. We also propose a model which allows to quantify the degree of functionalisation by using the individual Raman mode intensities. Furthermore, we demonstrate that while the coupling of the photoluminescence affects the intensity of the optical response in few-layered restacked homogeneous systems, the shift in peak position can be attributed to edge-to-edge and edge-to-basal plane interactions in (disordered) solvent-based systems and powders.

## Results

### Individual Raman intensities in monolayer graphene derivatives

**Figure 1:**
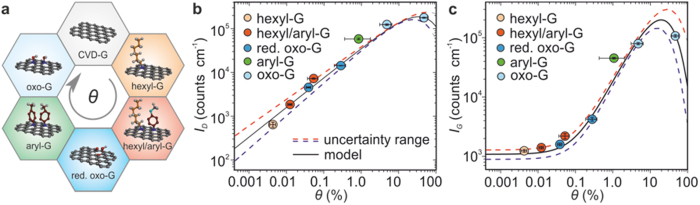
Photoluminescence modulated Raman intensity for different monolayer graphene derivatives. (**a**) Schematic illustrations of graphene derivatives with increasing *θ* clockwise from top: CVD-G, hexyl-G, hexyl/aryl-G, r-oxo-G, aryl-G, and oxo-G. (**b**) D- and (**c**) G-mode intensity, *I_D_* and *I_G_*, respectively, increase monotonically with *θ* despite having different types of functional addends. Solid lines correspond to the fitted model (Equations (2) and (3)) with dashed lines accounting for the uncertainty range.

We refer to covalently modified monolayer graphene obtained by different reaction routes (*cf.* Methods) to cover a wide range of *θ*. Figure 1a depicts the schematic representation of these derivatives: pristine CVD graphene (CVD-G), hexylated (hexyl-G)^24^, mixed functionalised (hexyl/aryl-G)^24^, reduced oxo-graphene (r-oxo-G)^25^,^26^, arylated (aryl-G)^27^ as well as oxo-graphene (oxo-G)^25^,^26^ (Supplementary note 1). In order to eliminate the impact of the addends on the optoelectronic response, the chemical moieties used were either non-Raman active^28^ or showed a Raman response far from the dominant graphene peak positions^29^.

The Raman spectra were taken at fixed experimental conditions (*cf.* Methods) and in the central areas of the flakes. For the purpose of this study, we focused on the dependence on the degree of functionalisation of the individual Raman D- and G-mode intensities, *I*_*D*_ and *I*_*G*_, respectively (defined as the integrated peak areas, *cf.* Supplementary note 2). While the positions of both peaks remained constant (*cf.* Supplementary Fig. 1), a continuous increase in both *I*_D_ and *I*_G_ with the basal C(sp^3^) centre content was observed (Fig. 1b and c), spanning several orders of magnitude. Although our samples were equipped with different moieties, remarkably no significant variation in neighbouring data points was found. This indicates that the specific Raman response is functionality-independent and driven by the sp^2^ domains. The PL intensity originating from these domains, *I*_*PL*_, can be described as^22^


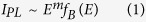


where *E* is the average band-gap of the sp^2^ domains (directly dependent on *θ*^23^), *m* an exponent associated with the density of states of the *π*-conjugated domains and *f*_*B*_*(E)* the Boltzmann distribution function. Following our initial assumption that the modulation of the optical response is a general effect, a specific coupling of the Raman signal to the PL can be expected, and thus the D-mode intensity to the lowest order can be written as


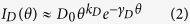


and the G-mode intensity


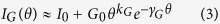


with *I*_*0*_ corresponding to the G-peak present in non-functionalised graphene. *D*_*0*_ and *G*_*0*_ account for the coupling strength of the D and G mode to the PL, respectively. It should be noted that all the parameters (including *k*_D_, *k*_G_, *γ*_D_, *γ*_G_) in equations (2) and (3) are dependent on the laser power, exposure time and temperature^23^. That is, a given set of parameter values is exclusively valid for a specific experimental condition during the Raman measurement. Therefore, the application of our model to a different experimental configuration requires a straightforward adjustment of the parameters in Eqs (2) and (3) (*cf*. Supplementary note 3).

The experimental data in Fig. 1b and c can be well reproduced within our model even considering the lower and upper uncertainty limits (*cf.* Table 1). This demonstrates that the experimentally observed Raman intensity increase can indeed be described as being modulated through PL-active sp^2^ domains.

### Raman intensity and *in situ* electrostatic doping of oxo-G

**Figure 2:**
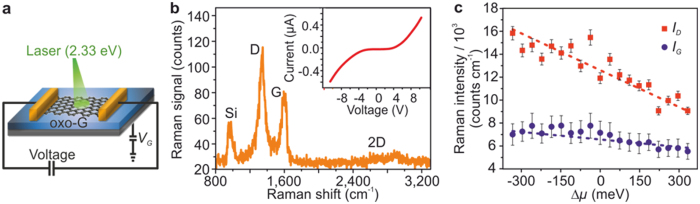
Change in Raman intensity with electrostatic doping of monolayer oxo-G (*θ* = 50%). (**a**) Schematic drawing of the measurement set-up combining *in situ* chemical potential shift with Raman spectroscopy. (**b**) Raman spectrum at zero gate-voltage. Inset: Current-voltage characteristic showing a symmetric non-linear dependence. (**c**) *I*_D_ and *I*_G_ as function of chemical potential shift Δ*μ*. During extraction of electrons the *I*_G_ intensity hardly changes, whereas the *I*_D_ intensity shows a significant dependence.

To provide further evidence of the coupling between PL and the Raman response, we studied the dependence of *I_D_* and *I_G_* on the *π*-state occupation for the case of oxo-G (*θ* ~ 50%) at room temperature. A single oxo-G flake^25^ was lithographically contacted with Ti/Au electrodes on a SiO_2_ (300 nm) substrate (*cf.* Supplementary note 4). The Si backside of the chip was used as gate electrode. By applying a gate voltage, *V*_G_, the charge density in the flake was varied by up to ±1.4 × 10^13^ cm^-2^ (*cf.* Supplementary note 5). This corresponds to a shift of chemical potential, Δ*μ*, by about ±370 meV, respectively. Raman spectra were taken at different gate voltages (schematic setup shown in Fig. 2a). This way, any optical transitions were directly tuned *via* electrostatic doping^30^ while preserving the structural integrity of the graphene.

In Fig. 2b, the Raman spectrum of oxo-G at zero gate voltage is shown. The evolution of the D- and G-mode intensities with varying chemical potential is shown in Fig. 2c. Extraction of electrons from oxo-G leads to a continuous increase of the D-mode intensity, which amounts to the total change of about 65% over the entire range of Δ*μ*. In contrast, the G-mode intensity is hardly varying with the chemical potential. The latter implies that no chemical reduction occurred in the material during the experiment since the G-mode intensity is proportional to the number of lattice C(sp^2^) atoms illuminated by the laser beam^30^. Noteworthy, considering an average *π*-conjugated domain size in oxo-G of approximately 1 nm^2^ 25,31, we can estimate that the addition of an extra electron will require 1 to 2 eV. This energy is of the same order of magnitude as the laser energy used (2.3 eV) implying that PL excitations are energetically possible. Also, this energy represents basically an activation energy which is well above the thermal energy at ambient conditions and thus consistent with the symmetric and nonlinear current-voltage characteristics^32^ (inset Fig. 2b). All these findings provide the final proof that the Raman intensity modulation originates from the photoluminescent C(sp^2^) domains, since electrostatic doping only changes the *π*-state occupancy.

### Comparison of the intensity-based model to existing approaches

**Figure 3:**
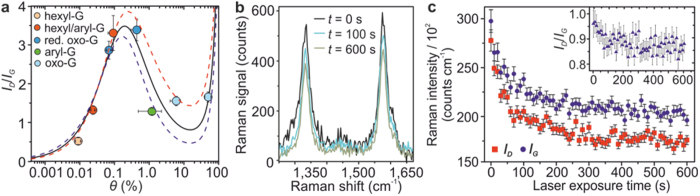
Correspondence between the intensity-based model with the *I*_*D*_*/I*_*G*_ approach for determining degree of functionalisation *θ*. (**a**) Ratio of the Raman modes, *I*_*D*_*/I*_*G*_, fitted with Eq. (2) and (3) (solid line, dashed lines correspond to the upper and lower uncertainty limits), recovering the mutual *θ*-dependence. Data points colour-coded for different addend types same as in Fig. 1b. (**b**) Change in Raman mode intensity during laser-induced defunctionalisation of hexyl-G. (**c**) *I*_D_ and *I*_G_ as function of laser-exposure time. The *I*_D_/*I*_G_ ratio is shown in the inset revealing a significantly smaller overall change with defunctionalisation than the individual intensities despite showing some sizable variation at the beginning of the defunctionalisation. In the limit of low *θ* (after long exposure times), the ratio becomes constant.

The consistency between our experimental data and our model shows that the functionalisation degree *θ* of a monolayer graphene can be unambiguously determined by solely relying on the actual intensities, *I_D_* and *I_G_*. Our model is also commensurate with the existing approaches which refer to the ratio of the Raman D- and G-modes, *I*_*D*_*/I*_*G*_, as a typical measure for the number of defects and disorder introduced^14^,^33^,^34^. This is demonstrated in Fig. 3a where the fitting of the *I_D_*/*I_G_* ratios with equations (2) and (3) recovers the mutual *θ*-dependence as reported in literature^14^,^33^,^34^. We note that for larger *θ* the uncertainty in the fitted *I*_D_/*I*_G_ ratios tends to increase, which suggests that in this regime higher-order terms in equations (2) and (3) might have to be taken into consideration.

We remark that the ratio *I_D_*/*I_G_* combined with the geometrical models is well known to be a limited description: First, no material-specific parameters are accounted for. Second, considering the addend related formation of *π*-conjugated domains^23^, this method only allows to deduce an average defect distance *L*_D_^14^,^33^,^34^ which is exclusively a good descriptive quantity for systems with a low amount of defects (well isolated basal C(sp^3^) centres). In other words, the *I_D_*/*I_G_* ratio is only suitable in the limit of a low *θ* (below ca. 1%). Third, no unique solution for *L*_D_ is provided. And fourth, *I*_D_/*I*_G_ leads to a loss of information which is contained in the individual *I*_D_ and *I*_G_ values and thus gives an incomplete measure regarding *θ*. This is illustrated in Fig. 3b,c for hexyl-G during laser induced defunctionalisation: With laser exposure time, both *I*_D_ and *I*_G_ values decreased by 35 to 40%. In contrast, the *I*_D_/*I*_G_ ratio was reduced by less than 8% (inset Fig. 3c). The use of our model therefore is beneficial over the current *I*_*D*_*/I*_*G*_ approaches.

### Raman spectra in multilayered systems

**Figure 4:**
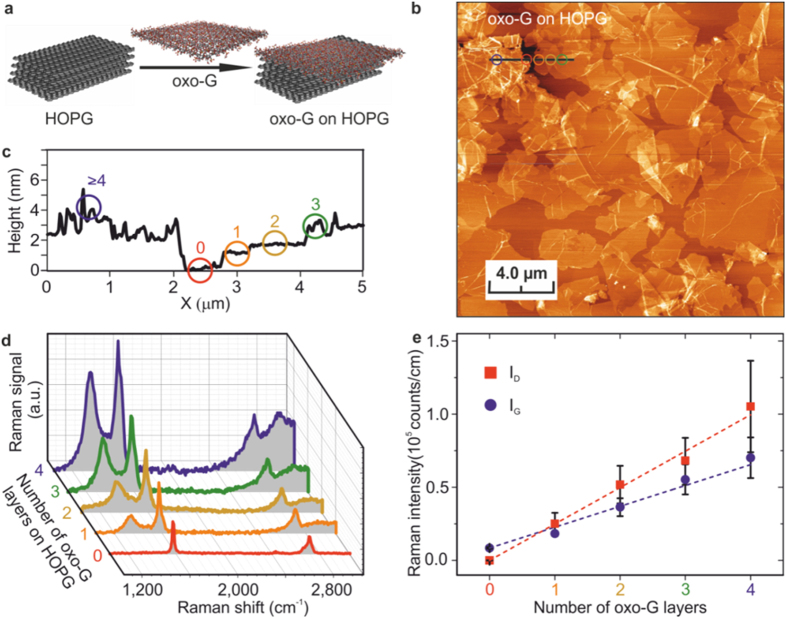
Evolution of the off-edge Raman response of stacked oxo-G layers with the layer number. (**a**) Schematic illustration of oxo-G layer deposition on HOPG. (**b**) AFM image of stacked oxo-G monolayers after deposition. (**c**) Height profile along the indicated line in (**b**). The colour-code indicates number of layers (red = 0; orange = 1; dark yellow = 2; green = 3, blue ≥ 4). (**d**) Representative Raman spectra for different number of layers. (**e**) Average *I*_D_ and *I*_G_ values for different number of layers showing a continuous increase with layer number. However, the different slopes indicate that the PL contributions are not purely additive but rather coupled to the Raman response.

Having established a direct link between PL-active sp^2^ domains and Raman intensity for individual monolayers of functionalised graphene, it is instructive to question how our findings translate to solution-processed bulk materials. In particular, we address the occurrence of a position shift in the optoelectronic response observed in powdered and solvent-based graphene derivative systems^1^,^16^,^17^,^18^. For this, we first chose regularly stacked oxo-G layers (*θ* ~ 50%) transferred on top of highly oriented pyrolytic graphite (HOPG) (Fig. 4a, *cf.* Methods). The Raman response was measured in the central areas (off-edge) of the flakes for different numbers of layers which were identified by AFM profile imaging (Fig. 4b,c). That is, here the areas close to the edges of the flakes were deliberately excluded from the analysis. With the used 532 nm laser excitation at 1 mW power, a penetration depth of at least 30 layers into the HOPG is expected based on the transmittance of single layer graphene^35^ (Supplementary Note 6). Representative Raman spectra for HOPG, mono-, bi-, tri- and ≥four-layer oxo-G are given in Fig. 4d showing an increase in signal intensities with each added layer, however, no shift in peak position was observed.

Accounting for the polydispersity of the system, we employed SRS^36^ to increase the statistical significance of the obtained data. From these measurements the average *I*_D_ and *I*_G_ values were extracted as function of layer number (Fig. 4e). For both, the peak intensities increase linearly, which shows that there are no significant interactions between adjacent oxo-G layers which might lead to the blocking of optical paths. However, the increments differ for *I*_D_ and *I*_G_, which indicates that the PL contributions in a stacked system are not purely additive. Therefore, for the very specific conditions of well-aligned, non-interacting systems with uniform functionalisation a simple extrapolation from single layer analysis to multi-layers using Raman spectroscopy should be feasible.

**Figure 5:**
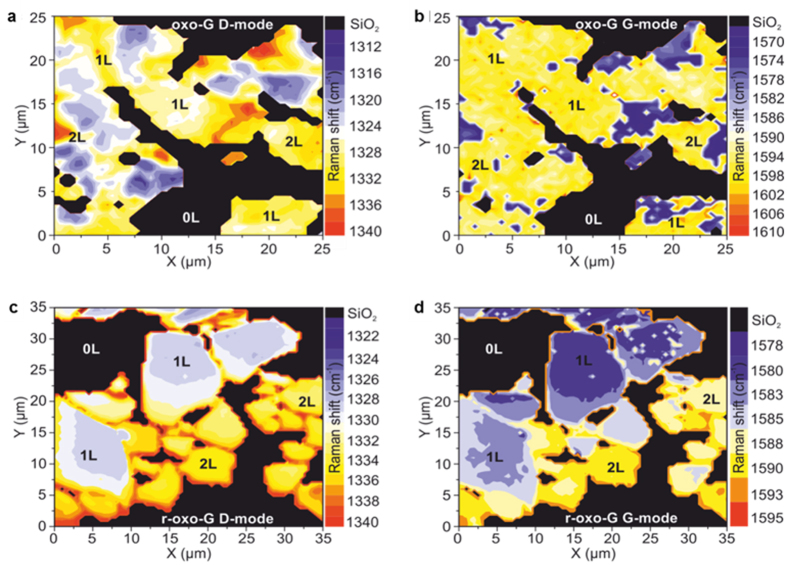
Dominant Raman mode positions in restacked functionalised layers on SiO2 substrate. (**a**) D-mode and (**b**) G-mode position map for a thin film of oxo-G (50%). (**c**) and (**d**) respectively show D- and G-mode position map for r-oxo-G (0.07%) Numbers of stacked layers are denoted on the graphs. In the case of oxo-G, the positions of the dominant modes remain approximately constant in the centres of the flakes, regardless of the layer number, while at the edges the positions shift towards higher values. An analogous observation is made in the case of r-oxo-G.

Importantly, the fixed position of the Raman peaks suggests the shift observed in non-aligned systems^1^,^16^,^17^,^18^ is not associated with the photoluminescent domains and is of another origin. To corroborate this, we studied the positions of the D- and G-modes in few-layer oxo-G and r-oxo-G films restacked on a SiO_2_ surface (*cf.* Methods). Figure 5a and b show Raman mode position maps of the D- and G-modes, respectively, for a oxo-G film (*θ* = 50%). Analogous data for r-oxo-G (*θ* = 0.07%) are shown in Fig. 5c and d. For oxo-G films no clear distinction of the mode positions between centre and edge regions could be identified due to the strong broadening of the individual Raman peaks. In contrast, while the *I*_*D*_ and *I*_*G*_ positions in the central area of the r-oxo-G flakes remain constant irrespective of the layer number, a distinct shift can be observed at and close to the flake edges. Considering that the oxo-G at *θ* = 50% has on average significantly smaller *π*-conjugated domains with associated stronger PL contribution at our laser lines than r-oxo-G at *θ* = 0.07%, the reported shift in position of peaks in the optical response of solvent-based and powdered functionalised graphene is primarily not associated with the sp^2^ domains. Therefore, it can be attributed to edge-to-edge and edge-to-layer (basal plane) interactions within such polydisperse systems.

## Discussion

Our combined study using Raman and electrostatic doping provides evidence that the generally observed modulation of the optical response in graphene derivatives is associated with photoluminescent sp^2^ domains, which form during chemical modification. Based on our findings, we demonstrate that the degree of functionalisation in covalently modified monolayer graphene can be directly related to the absolute Raman D- and G-mode intensities, and propose a phenomenological model which eliminates the ambiguity inherent to the geometrical *I*_D_/*I*_G_ ratio approaches, therefore making analysis of graphene derivatives less ambiguous. Furthermore, we show that a similar intensity-based approach should be feasible to quantify the defect density in the case of few-layered, well-ordered systems. However, the presence of edges, interlayer interactions and different stacking geometries in a (disordered) bulk or solvent-based system can lead to a position shift in the optical response. Our results also provide insight into the optical response of graphene quantum dots and assemblies of those where edge-to-edge interactions can play a role. Finally, the coupling of PL to Raman excitations can be expected to occur in other covalently functionalised layered 2D materials, too, and is probably even enhanced if they are inherently PL active unlike graphene.

## Methods

### Covalent functionalisation of graphene

Oxo-graphene (oxo-G) of 50% was synthesized from natural graphite in sulfuric acid with potassium permanganate as oxidant^25^; for 6% oxo-G the starting material was graphite sulphate^26^. Arylated CVD graphene (aryl-G): Bis-(4-*tert*-butylphenyl) iodonium hexafluorophosphate was deposited from solution (CH_2_Cl_2_) on a monolayer flake. The reaction of 4-*tert*-butylphenyl (^*t*^BP) cations was subsequently activated by a laser pulse (532 nm, 5 s, 10 mW) within the Raman spectrometer. Generated phenyl cations reacted with the carbon lattice of graphene by covalent bond formation^27^. The reaction scheme is shown in Fig. S1. Hexylated CVD graphene (hexyl-G), hexylated and arylated CVD graphene (hexyl/aryl-G): were synthesized according to Ref. 24. Laser-induced thermal defunctionalisation of hexyl-G was carried out with the laser line 532 nm and a power of 15 mW. The CVD graphene (*cf*. Supplementary Fig. 2 for Raman spectrum) was grown on Cu and purchased from Graphene Supermarket (https://graphene-supermarket.com/CVD-grown-graphene).

### Raman spectroscopy

Raman spectroscopic characterization was carried out on a Horiba Jobin Yvon LabRAM Aramis confocal Raman microscope (excitation wavelength: 532 nm) with a laser spot size of ~1 μm (Olympus LMPlanFl 100x, NA 0.90). Raman spectra were taken with a 532 nm laser line, 1 s acquisition time, 1 mW power, at room temperature. Statistical Raman measurements were obtained through a motorized x-y table in a continuous linescan mode (SWIFT-module).

### Preparation of Langmuir-Blodgett films

Langmuir-Blodgett Films of oxo-G on HOPG and SiO_2_ were prepared using Langmuir-Blodgett Minitrough from KSV NIMA. Oxo-G was dissolved in methanol/water mixtures. The subphase was water. Oxo-G transfer was carried out at the pressure of 10 mN m^−1^. Oxo-G was subsequently reduced by vapor of a 1:1 volume mixture of hydriodic acid and trifluoroacetic acid at 80 °C.

### Data availability

All relevant data are available from the authors on request.

## Additional Information

**How to cite this article:** Vecera, P. *et al*. Degree of functionalisation dependence of individual Raman intensities in covalent graphene derivatives. *Sci. Rep.* **7**, 45165; doi: 10.1038/srep45165 (2017).

**Publisher's note:** Springer Nature remains neutral with regard to jurisdictional claims in published maps and institutional affiliations.

## Supplementary Material

Supplementary Information

## Figures and Tables

**Table 1:** Values of the fitting parameters for D- and G-mode intensity obtained for the data in Fig. 3b and c.

D-mode		G-mode	
		*I*_0_	1084 ± 193
*D*_0_	28000 ± 3270	*G*_0_	11838 ± 1818
*k*_*D*_	0.73 ± 0.08	*k*_*G*_	1.34 ± 0.08
*γ*_*D*_	0.020 ± 0.009	*γ*_*G*_	0.06 ± 0.02
