# Supplementary material for: Degree of functionalisation dependence of individual Raman intensities in covalent graphene derivatives
Source: Sci Rep. 2017 Mar 27;7:45165. doi: 10.1038/srep45165 (PMC5366877; doi:10.1038/srep45165)
Supplement: Supplementary Information [file srep45165-s1.pdf]

Supplementary Information

Degree of functionalisation dependence of individual Raman intensities in covalent graphene derivatives

Philipp Vecera,[1] Siegfried Eigler,[1*] Maria Kolešnik-Gray,[2] Vojislav Krstić,[2*] Asmus Vierck,[3] Janina Maultzsch,[3] Ricarda A. Schäfer,[1] Frank Hauke,[1] and Andreas Hirsch[1*]

[1]*Department of Chemistry and Pharmacy and Joint Institute of Advanced Materials and Processes (ZMP), Friedrich-Alexander-Universität Erlangen-Nürnberg (FAU), Henkestrasse 42, 91054 Erlangen, Germany*

[2]*Chair for Applied Physics, Department of Physics, Friedrich-Alexander-Universität Erlangen-Nürnberg (FAU), Staudtstraße 7, 91058 Erlangen, Germany*

[3]*Institut für Festkörperphysik, Technische Universität Berlin, Eugene-Wigner-Building EW 5-4, Hardenbergstrasse 36, 10623 Berlin, Germany*

***Corresponding Authors:**

andreas.hirsch@fau.de, vojislav.krstic@fau.de, siegfried.eigler@fau.de

Supplementary note 1: Solvents and materials processing

Chemicals and solvents were purchased from Sigma Aldrich Co. (Germany) in HPLC quality and were used as-received if not stated otherwise. Tetrahydrofuran (THF) and 1,2-dimethoxyethane (DME) were received anhydrous from Sigma-Aldrich and dried over molecular sieves (3Å). Subsequently, they were distilled over Na/K alloy to remove the inhibitor and achieve absolute quality (<1 ppm H_2O, <1 ppm O_2). Finally, pump-freeze technique was used to completely degas the solvents prior to the reaction.

Sample preparation, solvent processing and functionalization reactions were carried out in an argon filled Labmaster SP glove box (MBraun), equipped with a gas filter to remove solvents and an argon cooling systems, with an oxygen content <0.1 ppm and a water content <0.1 ppm.

Supplementary note 2: Raman mode intensity *vs.* degree of functionalization

Raman spectroscopic characterization was carried out on a Horiba Jobin Yvon LabRAM Aramis confocal Raman microscope (excitation wavelength: 532 nm) with a laser spot size of ~1 µm (Olympus LMPlanFl 100x, NA 0.90). The measurements were carried out using a micro-Raman setup in backscattering geometry. The spectra were taken using a 532 nm laser line, 1 s acquisition time, 1 mW power, at room temperature.

Statistical Raman measurements were obtained through a motorized x-y table in a continuous linescan mode (SWIFT-module).

Principally a deconvolution of the Raman signal centered around 1580 cm^{-1} – 1620 cm^{-1} could be attempted. However, the experimental spectra revealed that such an approach is only feasible in a narrow range of degree of functionalization (0.05% to 0.5%). Therefore we referred to single Lorentzian fitting as analytical approach which carries a systematic error (accounted for in data error-bars), but has the advantage to cover the entire degree of functionalization range.

The degree of functionalization θ in the case of r-oxo-G, hexyl-G, aryl-G, and mixed functionalized hexyl/aryl-G was estimated using the I_D/I_G ratio approach[1-3]. Structural analysis of oxo-G was carried out *via* thermogravimetric and combustion analysis[4,5].

Supplementary note 3: guide for practical usage of model

There are two possible ways to apply the model: First, a calibration curve for the D-mode and the G-mode intensities for a specific experimental set-up/conditions can be generated. This includes, a fixed laser line, laser power, laser spot size, exposure time and environmental conditions. The work here provides the calibration curves (Fig. 1b and c) for graphene at the conditions 1 mW laser power, laser line 535 nm, spot-size 1 µm, exposure time of 1s at ambient conditions. The corresponding parameters for the model are given in Table 1. Second, since the D-mode intensity increases logarithmically with the degree of functionalization, it is sufficient to take two samples of known degree of functionalization. The only requirement is that the degree of functionalization should differ by at least one order of magnitude (*cf*. logarithmic dependence). With that the evolution of the D-mode intensity is fully defined for a set of experimental conditions.

We note that for these two approaches alternative ways of determining the degree of functionalization are needed. Nevertheless, for the limit of low degree of functionalization still the I_D/I_G ratio approach is suitable. However, since a I_D/I_G ratio of 1 is already reached at a degree of functionalization of around <3% (peaks become too broad), at higher degrees of functionalization other methods are necessary to determine the degree of functionalization. This is for example combustion elemental analysis or thermogravimetric analysis coupled with mass spectrometry.

Supplementary note 4: Electrical contacting of single layer oxo-G.

After deposition of oxo-G monolayers (θ = 50%) *via* Langmuir-Blodgett technique on SiO_2 (300 nm)/Si(n^{++}) substrate, the definition of electrodes was carried out by electron-beam lithographical means (Zeiss Supra 40 equipped with Elphy lithography

attachment) and followed by e-beam deposition of the contact material (Ti/Au; 5 nm/30 nm). The highly doped Si backside served as global backgate (shift in carrier-density/chemical potential).

Supplementary note 5: *In situ* Raman and electrostatic gating.

To facilitate *in situ* measurements, the samples were transferred onto commercially available chip-carriers and wire-bonded, and then inserted into a dedicated socket located directly in the laser path of the Raman spectrometer. The socket was connected to a standard electrical characterization set-up. Assuming the π-conjugated C(sp^2) domains to be 2D metallic discs, based on the self-capacitance given by $C_{self} = 8\varepsilon_0\varepsilon_r R$ (radius $R \approx 0.5$ nm, ε_0 the vacuum permittivity, $\varepsilon_r \approx 3.5$ for the C(sp^3) matrix), the charging energy e^2/C_{self} (e – elementary charge) can be estimated to be about 1.4 eV.

Supplementary note 6: Increasing of intensities with oxo-graphene deposition on HOPG.

Oxo-graphene principally consists of π-conjugated domains whose electronic confinement gives rise to photoluminescent activity. In stark contrast, in pure graphene (local) confinement of similar type does not exist and therefore no photoluminescence contributions which could enhance the Raman response are present. Therefore, the Raman signal intensity of a oxo-graphene monolayer can easily become comparable to the signal response of several underlying layers of HOPG graphene. Consequently, a stack of few-layers of oxo-graphene can even “hide” fully the signal of the underlying layers.

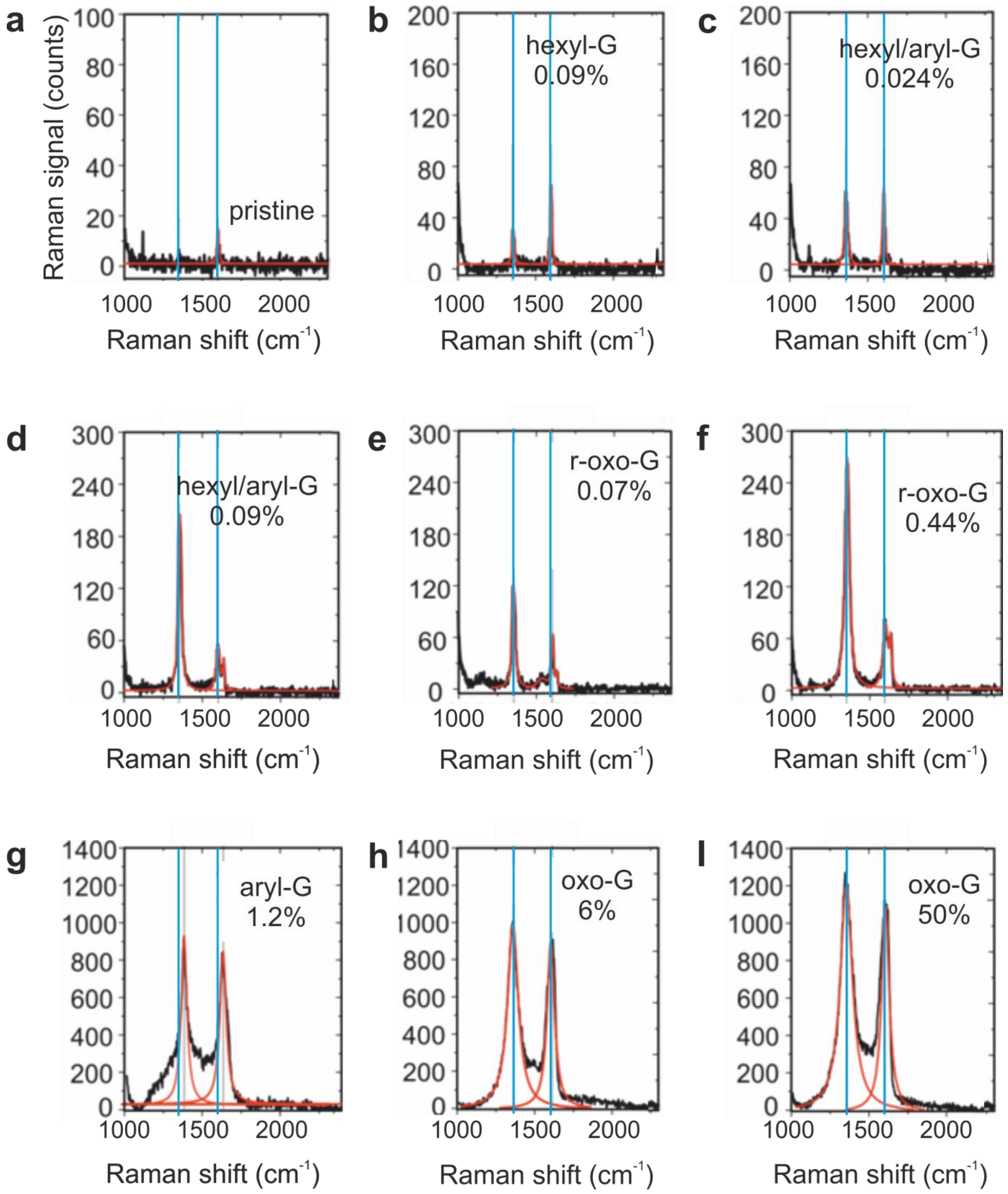

Supplementary Figure 1. Raman spectra of differently functionalized graphene derivatives: (**a**) pristine CVD graphene, (**b**) hexylated graphene, (**c**), (**d**) double functionalized hexylated and arylated graphene, (**e**), (**f**) reduced oxographene, (**g**)

arylated graphene and (**h**),(**i**) oxographene. Degrees of functionalization of each derivative are denoted in graphs. Red lines show the Lorentzian fits used to determine the peak area. Blue lines denote the constant positions of the D and G modes.

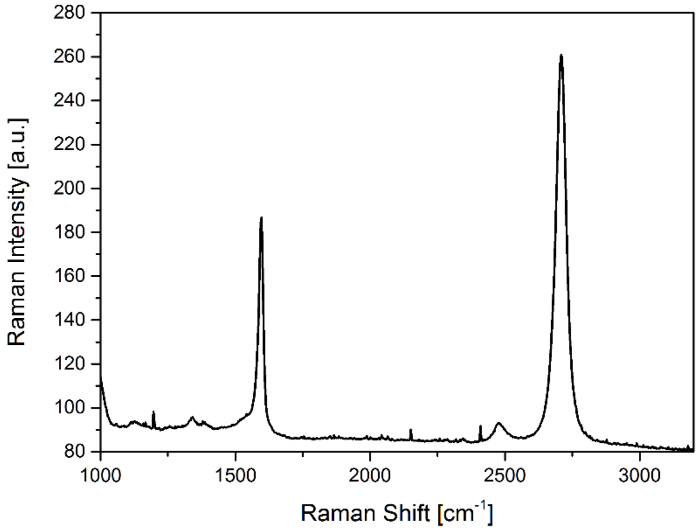

Supplementary Figure 2. Extended Raman spectra of pristine CVD graphene.

References

1. Lucchese, M. M. *et al. Carbon* **48**, 1592-1597 (2010).
2. Cançado, L. G. *et al.* *Nano Lett.* **11**, 3190-3196 (2011).
3. Englert, J. M. *et al.* *ACS Nano* **7**, 5472-5482 (2013).
4. Eigler, S. *et al. Adv. Mater.* **25**, 3583-3587 (2013).
5. Eigler, S. *Chem. Commun.* **51**, 3162-3165 (2015).
